# Nerve fibre organisation in the human optic nerve and chiasm: what do we really know?

**DOI:** 10.1038/s41433-024-03137-7

**Published:** 2024-06-07

**Authors:** Pratap R. Pawar, Joshua Booth, Andrew Neely, Gawn McIlwaine, Christian J. Lueck

**Affiliations:** 1 School of Engineering and Technology, University of New South Wales, Canberra, NSW Australia; 2 School of Medicine and Psychology, Australian National University, Canberra, NSW Australia; 3 Department of Ophthalmology, Mater Hospital, Belfast, Northern Ireland

**Keywords:** Retina, Object vision

## Abstract

A recent anatomical study of the human optic chiasm cast doubt on the widespread assumption that nerve fibres travelling in the human optic nerve and chiasm are arranged retinotopically. Accordingly, a scoping literature review was performed to determine what is known about the nerve fibre arrangement in these structures. Meta-analysis suggested that the average number of fibres in each optic nerve was 1.023 million with an inter-individual range of approximately 50% of the mean. Loss of nerve fibres with age (approximately 3,400 fibres/year) could not account for this variability. The review suggested that there might be a retinotopic arrangement of nerve fibres in the orbital portion of the optic nerve but that this arrangement is most likely to be lost posteriorly with a more random distribution of nerve fibres at the chiasm. Limited studies have looked at nerve fibre arrangement in the chiasm. In summary, the chiasm is more ‘H-shaped’ than ‘X-shaped’: nerve fibre crossings occur paracentrally with nerves in the centre of the chiasm travelling coronally and in parallel. There is interaction between crossed and uncrossed fibres which are widely distributed. The review supports the non-existence of Wilbrand’s knee. Considerable further work is required to provide more precise anatomical information, but this review suggests that the assumed preservation of retinotopy in the human optic nerve and chiasm is probably not correct.

## Abstract (zh)

最近一项关于人类视交叉的解剖学研究对人视神经和视交叉中的神经纤维是否以视网膜区域定位的方式排列的普遍接受的观点提出了质疑。因此, 我们对文献综述进行了范围界定, 以确定这些结构中神经纤维排列的已知信息。Meta分析表明, 每条视神经中的平均纤维数量为102.3万条, 个体间差异约为平均值的50%。随年龄增长造成神经纤维的丢失(大约3400根/年)不能解释这种可变性。本综述提出, 视神经的眼眶部分可能存在神经纤维的视网膜定位排列, 但随后这种排列方式很有可能在视交叉处随着神经纤维的随机分布而消失。总之, 视交叉更多的是‘H’形而不是‘X’形: 神经纤维交叉出现在中央附近, 视交叉中心的神经沿冠状方向平行行进。广泛分布的交叉纤维和非交叉纤维之间存在相互作用。本综述支持Wilbrand膝不存在的说法。要获得更精确的解剖学信息还需大量的进一步研究, 但本综述提出, 在人类视神经和视交叉中视网膜区域对应的假设可能并不正确。

## Introduction

It is widely accepted that compression of the optic chiasm by an expanding pituitary tumour typically gives rise to bitemporal visual loss [1]. While the extent of the visual field loss in the two eyes may be variable [2], the consistent clinical feature is a midline vertical step: vision on the nasal side of the step is relatively preserved compared with that on the temporal side. This implies that fibres arising from the nasal retina (i.e. those serving the temporal visual field and destined to cross to the contralateral optic tract and hemisphere) are more vulnerable to damage than the uncrossed fibres arising from the temporal retina that project ipsilaterally. To date, no entirely satisfactory explanation for this increased vulnerability exists, but several theories have been proposed. These have suggested that crossing fibres could be selectively damaged by stretch [3], pressure [4], or change in blood supply [3, 5]. More recently, it has been proposed that it is the nerve fibre crossing itself that confers the increased vulnerability to compression [6]: any compressive force on the chiasm will be spread over a smaller area of nerve-to-nerve contact between crossing fibres, thereby generating a greater concentration of stress and, hence, damage to those fibres.

**Fig. 1:**
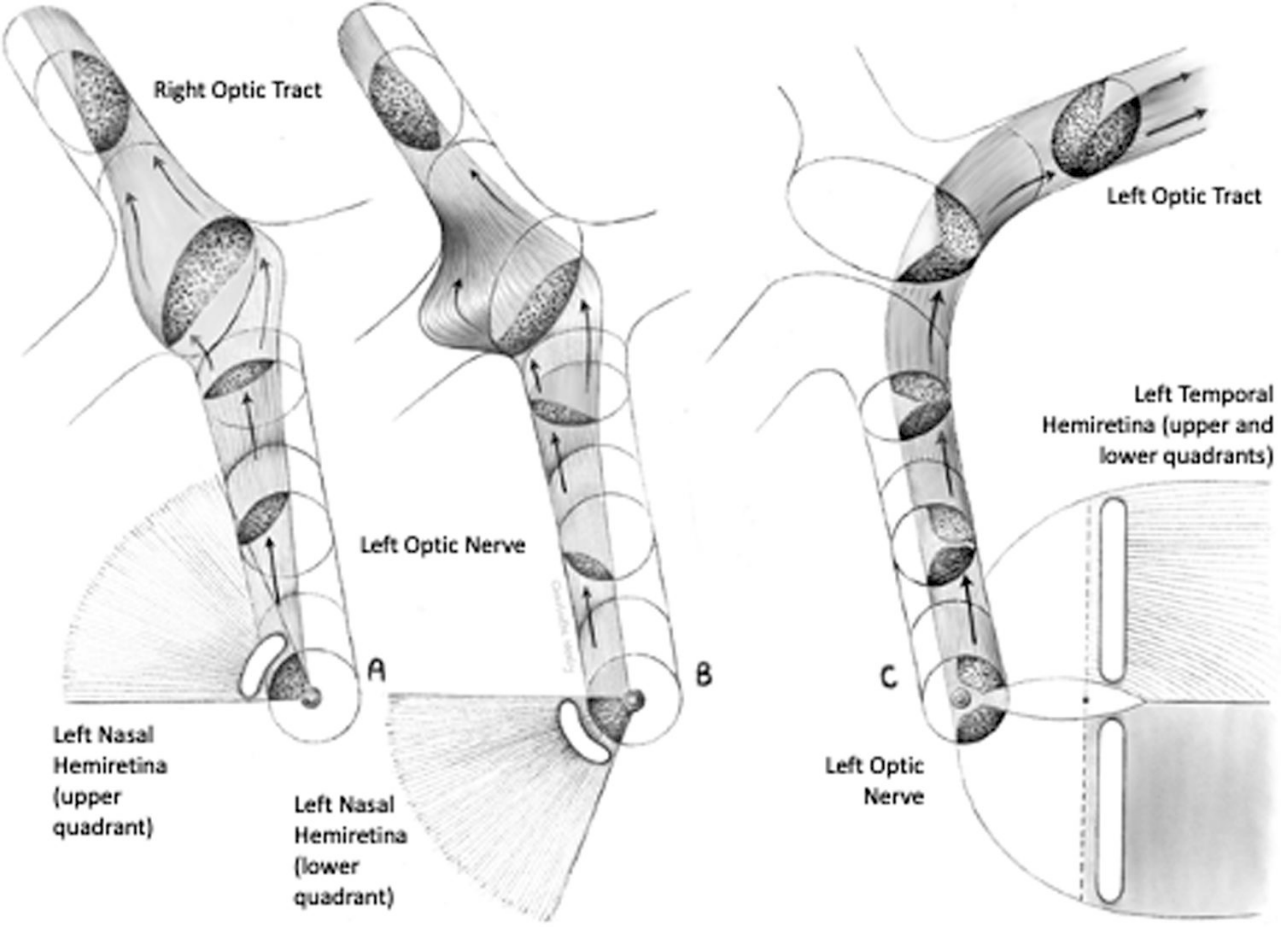
Summary diagram representing the current concept of retinotopic organisation of nerve fibres in the anterior visual pathway. **A**, **B** Fibres from the upper (**A**) and lower (**B**) parts of the nasal hemiretina cross centrally in the chiasm to reach the contralateral optic tract. **C** uncrossed fibres from the temporal hemiretina pass directly to the ipsilateral optic tract (after [9]). (Note the representation of Wilbrand’s knee in B is now thought to be artefact.).

It is not possible to test this ‘crossing’ theory in vivo so an in silico experiment looking at chiasmal compression was recently undertaken using finite element modelling [7]. The results were very supportive of the ‘crossing hypothesis’ [6]. Development of this model necessitated making various assumptions, including an assumption about how the nerve fibres within the chiasm were anatomically arranged. The model was constructed on the widely accepted concept of ‘retinotopic’ organisation of nerve fibres in the anterior visual pathways [Fig. 1], namely that fibres from the nasal half of the retina travel backwards (in parallel with each other) in the medial portion of the optic nerve to reach the optic chiasm. These fibres then cross homologous fibres arriving from the contralateral nasal retina in the centre of the chiasm before leaving the chiasm posteriorly in the contralateral optic tract (again, in parallel) [8–10]. Fibres from the temporal retina, on the other hand, pass directly backwards in the lateral part of the optic nerve, transiting through the lateral portion of the chiasm before exiting in the lateral part of the ipsilateral optic tract without having crossed. These uncrossed fibres are assumed to remain roughly parallel to each other throughout their course without interacting with any of the crossing fibres [Fig. 1] [10].

While developing the finite element model [7], it became clear that considerably more anatomic detail was required at a microscopic level. A subsequent photomicroscopic study of the chiasm [11] revealed several disparities with the conventional understanding described above. Most obviously, most individual nerve fibre crossings were found to occur paracentrally rather than in the midline. In the centre of the chiasm, nerve fibres were largely arranged in parallel, running in a transverse (i.e. left-right) direction. As expected, most fibres in the lateral parts of the chiasm travelled in an antero-posterior direction but the boundary with the crossing fibres was not clearly demarcated and there were many low-angle nerve fibre crossings in the lateral regions. These findings meant that the assumptions on which the original finite element model had been based were probably not valid. Not only that, they also called into question the widely accepted assumption of a retinotopic arrangement in the anterior visual pathway.

Accordingly, a scoping review of the literature was undertaken to determine precisely what is known about the arrangement of nerve fibres in the anterior (pregeniculate) visual pathway with particular reference to retinotopic organisation of the human optic nerve and chiasm. Particular questions to be addressed included:

1. how many nerve fibres travel in the human optic nerves and chiasm?
2. to what extent are the nerve fibres in the human optic nerves and chiasm retinotopically organised?
3. which nerve fibres cross each other in the chiasm, and where do they cross? How many times do individual nerve fibres cross, and at what angle do they cross at each of these crossings?
4. what nerve fibres other than those arising from retinal ganglion cells and destined for the lateral geniculate nuclei (LGN) travel through the optic chiasm?

## Methods

**Fig. 2:**
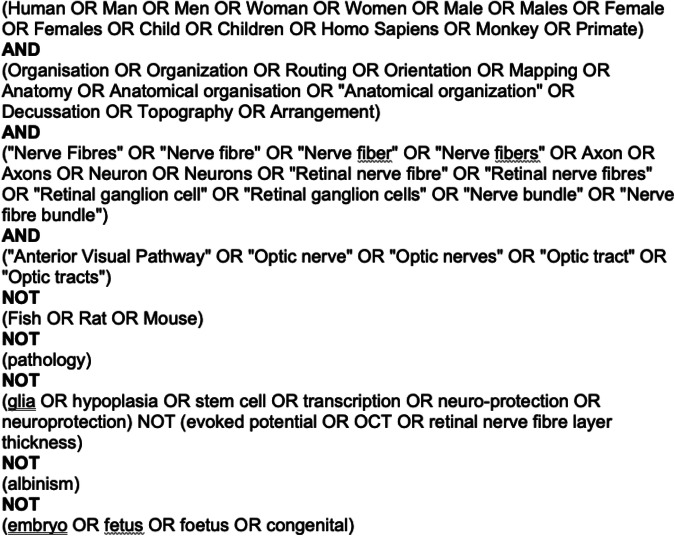
Search strategy used for the literature review.

**Fig. 3:**
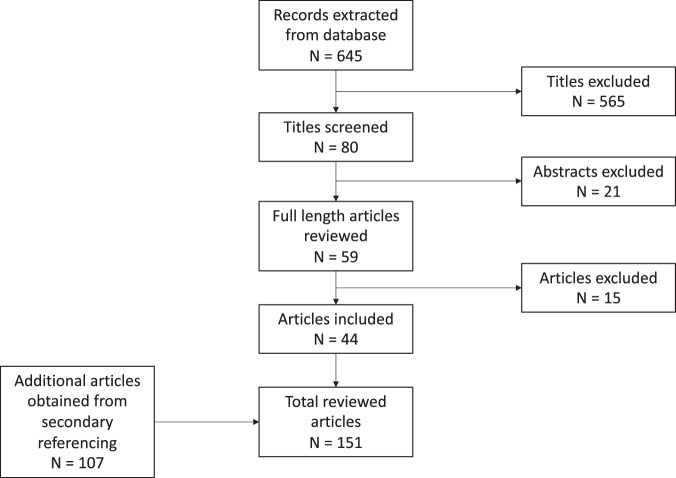
PRISMA diagram detailing selection of articles included in the review.

The review was registered on PROSPERO (PROSPERO 2021 CRD42021247266). A search was performed and updated in November, 2023, on PubMed, Medline and Google Scholar using the strategy provided in Fig. 2, looking for relevant publications. Key words referred to synonyms of primate species, nerve fibre organisation and anterior visual pathway structures. Because this search was designed to look at normal human anatomy, terms such as ‘pathology’, ‘stem cells’, and ‘embryo’ were deliberately excluded. Duplicates were excluded from the initial search. Extracted titles and abstracts were reviewed for inclusion by two of the authors and any disagreement was resolved by mutual discussion. Articles were retained if they included information specifically related to the nerve fibre arrangement in human optic nerve and/or chiasm. In addition, articles relevant to more general considerations (e.g. a general description of inter-species differences, or the presence of unmyelinated or retrograde fibres in the optic nerve and/or chiasm) were also retained. Considerable secondary referencing revealed a number of other articles relevant to more general consideration (e.g. historical aspects) which have also been included in this review. Given the relatively large numbers of papers obtained through secondary referencing, the study was considered to represent a ‘scoping’ review rather than a ‘systematic’ review. A PRISMA diagram is provided in Fig. 3.

## Results

### The optic nerve

#### Optic nerve fibre numbers in humans

Macroscopically, the optic nerve leaves the eye at the lamina cribrosa and passes backwards through the orbit, the optic canal, and the intracranial subarachnoid space to reach the optic chiasm. Immediately behind the optic disc, the optic nerve fibres are arranged in about 500 fascicles separated by pial septa [8]. These septa are made up of connective tissue which is continuous with that of the pia mater surrounding the optic nerve [12, 13]. The septa persist throughout the orbital portions of the optic nerves but are gradually lost in its intracranial portions and have completely disappeared by the time it reaches the optic chiasm [14–16]. It has been suggested that the septa contribute to maintaining the integrity and flexibility of the orbital portions of the optic nerves in light of the extensive demands generated by eye movements [12, 13].

The nerve fibres in the human optic nerves are smaller and have thinner myelin sheaths than those in other cranial and peripheral nerves [17]. Fibre diameters range from 0.8-10 μm, though the vast majority of fibres have diameters of 1–2 μm. Progressively larger fibres are found peripherally [18]. Though discounted in the first half of the 20th century [19, 20] the optic nerves do contain small numbers of unmyelinated nerve fibres whose diameters range from 0.1–1.3 μm [17, 21]. These fibres are found predominantly around the central retinal artery: some of them are probably vasomotor but, overall, unmyelinated fibres are considered unlikely to be involved in transmitting visual information.

It is generally accepted that there are approximately 1 million (myelinated) nerve fibres in each human optic nerve [1, 8]. Since the late 1800s, many investigators have attempted to determine the precise number using different histological techniques and different methods of counting. The total number of nerve fibres has usually been determined by counting the fibres in each of a small number of regions of interest and then multiplying up to provide a total overall estimate. There is considerable inter-individual variation between the numbers published in the various studies [Table 1], individual nerve fibre numbers ranging from a minimum of around 400,000 [22–24] to a maximum of over 1,500,000 [25–27]. Mean values from the various studies have ranged from 0.5 million [28] to 1.2 million [25, 29] but inter-individual variation has often exceeded 50% of the mean within individual studies [27, 30–32]. Parenthetically, one possible explanation for this inter-individual variability might be that it reflects individual differences in embryogenesis: nerve fibre numbers are thought to peak at 3.7 million at 16–17 weeks of gestation with a subsequent loss of over 70% of fibres by 29 weeks to around 1.1 million: [33, 34] the proportion of axons lost during this process might vary from person to person.

**Table 1:** Studies which have estimated the number of nerve fibres in the human optic nerve.

Date	Authors	N	Age	Fixation	Mean (± Standard deviation)	Min	Max
1876	Krause [162]^a^	–	–	–	1,000,000	–	–
1879	Kuhnt [163]	2	–	silver	40,000	–	–
1880	Salzer [23]	3	–	Osmium/Müller’s liquid	437,645	413,000	465,558
1880	Krause [22]	–	–	–	440,347	–	–
1915	Zwanenburg [24]^a^	1	–	Weigert–Pal	440,000 [56]–754,623 [164]	–	–
1934	Arey & Schaible [30]^b^	2	–	silver	1,024,000/ 1,007,500	761,000/ 697,000	1,287,000/ 1,318,000
1935	Arey & Bickel [19]^b^	6	>1	silver	1,304,306	1,225,666	1,435,543
1940	Bruesch & Arey [28]^b^	–	–	silver	564,776–1,140,000	–	–
1942	Bruesch & Arey [20]	10	47–70	silver	1,010,000 (84,000)	871,000	1,200,000
1963	Oppel [56]	1	30	Weigert–Pal	1,186,172	–	–
1967	Kupfer et al. [81]	1	78	silver	1,150,227	–	–
1972	Potts et al. [39]	2	20 –35	toluidine blue	–	1,163,100	1,273,802
1982	Quigley et al. [164]	5	58–86	*p*-phenylene diamine	963,932	922,232	1,040,763
1984	Balazsi et al. [25]	16	3.5–82	*p*-phenylene diamine	1,244,005 (20,033)	911,900	1,645,490
1987	Johnson et al. [26]	13	31–86	*p*-phenylene diamine	1,084,000	759,000	1,610,000
1989	Repka & Quigley [36]	19	4–84	toluidine blue	693,316 (110,387)	519,662	904,086
1989	Mickelberg et al. [31]	12	35.9	*p*-phenylene diamine	969,000 (239,000)	655,000	1,355,000
1990	Jonas et al. [27]	22	20–75	toluidine blue	1,159,000 (196,000)	816,000	1,502,000
1992	Jonas et al. [32]	56	19–88	toluidine blue	1,158,000	777,000	1,679,000
2002	Cavallotti et al. [29]	16	18–22	toluidine blue	1,209,382^c^	–	–
2002	Cavallotti et al. [29]	34	68–76	toluidine blue	900,480^c^	–	–

^a^original articles could not be obtained: information derived from other references [31, 39, 164].

^b^abstract only.

^c^extrapolated from data in table (axons/mm^3^ x optic nerve area).

Technical issues may have contributed to some of the reported interindividual differences. For example, the number of axons falls with increasing time to fixation [25, 31], one study suggesting a rate of 13,521 axons/hour [35]. Though it has been suggested that this effect is not significant if fixation occurs within 9.5 h [32], it might have contributed to some of the variability between studies. Other technical sources of variability include possible shrinkage during fixation and the relatively small proportion of the whole nerve represented by the sampled regions of interest [36].

Another factor which might have contributed is aging. With age, the interfascicular septa in the optic nerve thicken and the individual nerve fibres demonstrate increasing numbers of corpora amylacea, gliosis and demyelination [37]. Many studies have found that nerve fibre numbers decrease with age but, again, the precise values vary between studies. One study suggested that fibre loss occurred at a rate of 500 axons/year [36] but most other studies have suggested an annual loss of around 4000–5000 axons/year [25, 27, 29, 31, 32, 35], a figure which is, incidentally, consistent with that derived from a study using high-resolution perimetry [38].

**Fig. 4:**
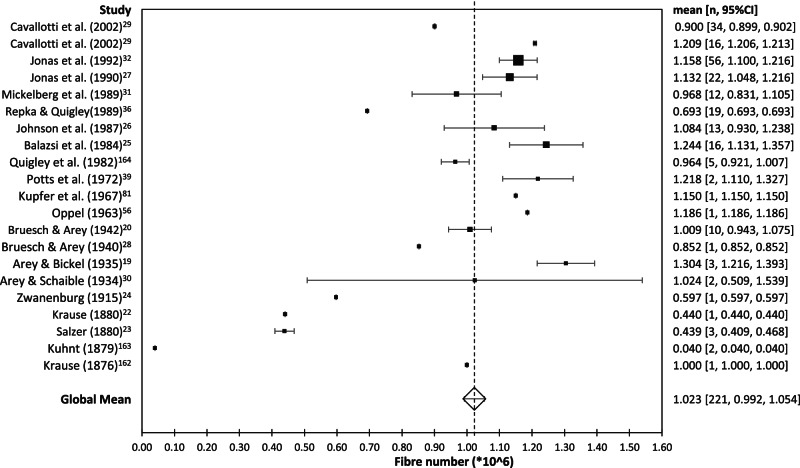
Forest plot showing total number of fibres in optic nerves as a function of publication date.

**Fig. 5:**
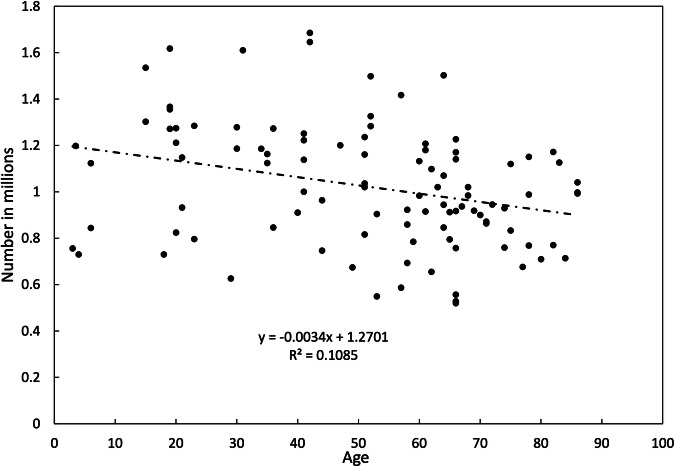
Optic nerve fibre numbers as a function of age.

Because any technical issue might have applied to any of the published studies, a meta-analysis of all published studies was performed. In addition to looking at the total number of fibres per nerve, additional analyses looked at whether there was an effect of publication date, staining technique, age and/or sex. The results are shown in Figs. 4 and 5.

In all, data from 221 individual measurements in 21 papers or abstracts were available. Meta-analysis of all studies yielded a global mean of 1.023 million fibres per optic nerve with a 95% confidence interval of ± 310,000 fibres. As shown in Fig. 4, a few of the very early studies yielded lower values but the number of individuals involved in these studies was very small. Otherwise, there was no obvious effect of year of publication. Only two studies used the Weigert-Pal staining technique making interpretation difficult. Toluidine blue (*N* = 92) and paraphenylene diamine (*N* = 34) yielded slightly higher overall numbers than silver stains (*N* = 20) but any differences attributable to different stains were not significant. Regression of individual nerve fibre numbers against age (where this information was provided) suggested an average overall rate of nerve fibre loss of 3400 fibres/year but, again, this did not reach significance [Fig. 5]. Information about sex was provided for 48 individuals: nerve fibre numbers were about 2% higher in males but, again, this difference was not significant.

#### Comment on comparative anatomy relating to nerve fibre numbers

Large numbers of studies have looked at the optic nerves of many different species. Unfortunately, the placement of the eyes and the anatomy of the anterior visual pathways of most of these animals differs significantly from those of humans meaning that any findings cannot be reliably extrapolated to humans. Studies on non-human primates have suggested that their optic nerves contain somewhat higher numbers of fibres than those of humans: in macaques, studies have found values ranging between 1.1 and 1.8 million [33, 39–42] and a figure of 1.8 million has been found in baboons [43]. As in humans, there is considerable inter-individual variation, with ranges of approximately 50% of the mean [41]. Looking at the effects of aging, one study found an average loss of 4300 nerve fibres/year but this figure did not reach significance either [41].

#### Retinotopic nerve fibre organisation in the human optic nerve

**Fig. 6:**
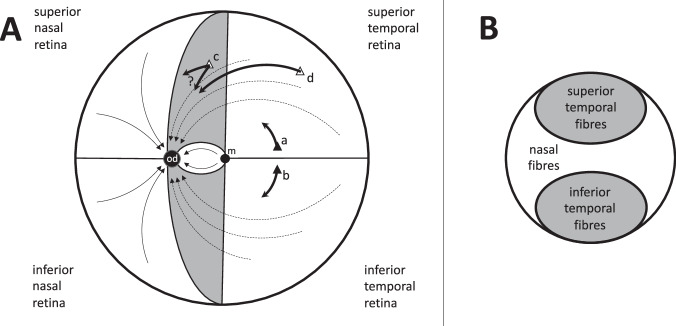
Diagram showing the course of optic nerve axons arising from retinal ganglion cells (RGCs) in different regions of the retina. **A** Axons from RGCs in the macula (m) pass directly to the optic disc (od) via the papillomacular bundle; axons from the superior temporal retina enter the disc superiorly while those from the inferior temporal retina enter it inferiorly; axons from the nasal retinal quadrants enter the optic disc via the upper and lower parts of its medial border [49]. Temporal RGCs which are contiguous but just above (a) and below (b) the temporal raphe (the horizontal meridian) will therefore send their respective axons to diametrically opposite poles of the optic disc, meaning that preservation of a precise retinotopic map of the retina in the optic nerve is not possible. The precise pathway of axons arising from RGCs in the shaded areas (i.e. those parts of the retina which are nasal to the macula but still temporal to the optic disc) is not clear. If axons from an RGC in this part of the retina (c) pass directly to the optic disc, they must travel alongside axons arising from RGCs in the superior temporal quadrant (d), again making a retinotopic map in the optic nerve impossible. Alternatively, if axons from these RGCs pass nasally across temporal fibres to enter the optic disc from its nasal aspect, this would mean that axons crossed each other in the retina, an arrangement for which there is currently no evidence [49]. **B** Diagram showing resulting nerve fibre arrangement in a cross-section of the anterior portion of the optic nerve: [59]: at this point fibres from temporal RGCs travel in two bundles (shaded) separated by fibres arising from nasal RGCs.

As above, the currently accepted concept is that adjacent nerve fibres travelling along an optic nerve serve immediately adjacent portions of the visual field and that this relationship is preserved throughout the optic nerves, through the chiasm and on into the optic tracts. Intuitively, a retinotopic organisation seems quite possible at the origin of the optic nerve where adjacent fibres appear to enter it from adjacent retinal locations [Fig. 6] [44]. However, this does not necessarily mean that a retinotopic arrangement is preserved along the entire length of the optic nerve or on into the chiasm and optic tract. Indeed, Melling et al. [15]. have published electron microscopic evidence of nerve fibres intermingling with each other in the posterior part of the human optic nerve.

At this point it is important to point out that a cross-section of the optic nerve could not represent an absolutely precise topographic map of the retina as is often suggested [45–50]. As shown in Fig. 6, nerve fibres entering the optic disc from the retina are configured such that fibres from the macula travel directly to the temporal rim of the optic disc in the papillomacular bundle [44]. Fibres entering the optic disc above and below this bundle are derived from the superior and inferior parts of the temporal retina, respectively, while fibres entering via the nasal portion of the optic disc are derived from the remaining nasal retina [44]. It is this arrangement which is responsible for the pathological development of bowtie, or band, atrophy of the optic disc [51–53] and optic nerve [54] when there is selective damage to nasal (crossing) nerve fibres. This means that there is an area of ‘retinotopic uncertainty’ relating to those fibres arising from the vertical strip of retina lying between the optic disc and the macula. If a nerve fibre arising in this region travelled directly to the optic disc, it would be travelling next to fibres arising from non-contiguous areas of the temporal retina. Similarly, fibres from temporal ganglion cells lying on either side of the horizontal meridian actually enter the optic disc at opposite poles [Fig. 6A]. Consistent with this arrangement, a histological study of a human optic nerve following longstanding damage to crossing fibres in the chiasm demonstrated that fibres arising from the temporal retina were arranged in two clear oval-shaped bundles in the superior and inferior portions of the optic nerve [54] [Fig. 6B].

The origin of the concept of retinotopy in the optic nerve is of interest. The arrangement of fibres in the optic nerves was first studied in the late 19th and early 20th centuries by many researchers, including Holmes and Lister in England, Pierre Marie and Chatelin in France, and Axenfeld and others in Germany [55]. These investigators studied non-human primates and used various stains to detect anatomically localised areas of optic nerve fibre damage following selective lesions made to the retina. The retinal lesions were initially made surgically but photocoagulation was used once this technique became available [10]. Stains used to detect nerve fibre degeneration included the Weigert-Pal [24, 56], Marchi [55, 57, 58] and Nauta [10] techniques before silver, toluidine blue and paraphenylene diamine stains were more widely adopted [Table 1]. In addition, a small number of complementary studies used a different approach, specifically looking at the retrograde distribution of label in the retina and/or optic nerve following injection of horseradish peroxidase into the optic tract or LGN [59, 60].

These studies led to considerable initial debate regarding whether or not the primate optic nerve was retinotopically organised. Henschen [61] and Pick [62] first suggested a retinotopic arrangement in the 1890s and this concept subsequently gained support from other studies such as those looking at the effects of lesions of the papillomacular bundle [9]. When looked at objectively, though, the published images from these early studies show that, while degenerating nerve fibres were indeed concentrated in one part of the optic nerve following localised damage to the retina, they were not completely restricted to a single location. Indeed, Parsons questioned the concept of retinotopy as early as 1902, having observed ‘scattered degenerated fibres throughout the optic nerve’ [58]. At least some of the observed scatter in the early studies may have been the result of accidental damage to nerve fibres arising from locations other than the intended retinal lesions [10] but this would not explain the considerable overlap observed in damaged nerve fibres resulting from lesions made in quite disparate parts of the retina, as described in some studies [63].

In retrospect, it is possible that some of the confusion might be explained by what is now known about the changes in the microscopic anatomy of the optic nerve as it passes backwards within the skull. Specifically, the septa are no longer present in the intracranial part of the nerve so it becomes much easier for the nerve fibres to mingle at that point [15]. Hence, it is possible that any retinotopic arrangement of nerve fibres present in the more anterior parts of the optic nerve is progressively lost posteriorly, and certainly by the point at which the nerve joins the chiasm.

#### Comment on comparative anatomy relating to retinotopic nerve fibre arrangement

As above, the optic nerves have been studied in many different animals. Many of these studies have supported the concept of a retinotopic arrangement, at least in the orbital portions of the nerves. However, there is considerable inter-species variation. For example, it is clear that there is no mixing of crossed and uncrossed fibres at all as the fibres reach and pass through the chiasm in species such as the quokka (a marsupial) and the tree shrew [64, 65], but a recent study in a non-human primate [66] found that crossed and uncrossed nerve fibres from the two eyes were completely intermingled in the optic tracts just distal to the chiasm. In his comprehensive review, Jeffery [67] concluded that there was a retinotopic arrangement of nerve fibres along most of the length of the orbital optic nerves of many different species, including rodents [68], marsupials [69], ferrets [70], cats [71, 72], monkeys [73], and humans [10]. However, in many of these species this retinotopic arrangement was lost posteriorly near the chiasm, around the point at which the septated architecture disappeared, meaning that the nerve fibres could, and did, intermingle [68]. Interestingly, electron microscopic studies of the septa have found that they contain many round pores, thought possibly to enhance the flexibility of the optic nerves [12]. These pores might allow nerve fibres to begin to mingle, at least to some extent, in the orbital portion of the optic nerve, but unrestricted intermingling becomes much more likely once the septal architecture is lost posteriorly.

Other evidence arguing against a retinotopic arrangement in the posterior portions of the optic nerves comes from the studies of retrograde injections of HRP into the LGN in the macaque referred to above. These demonstrated increasing nasotemporal scatter of nerve fibres in the optic nerves as they progressed backwards from the eye towards the chiasm [59, 60]. This observation is consistent with neurophysiological recording from adjacent neurons in the optic nerve of a spider monkey which revealed that the nerves’ receptive fields were randomly distributed, not adjacent [74].

#### Summary: nerve fibre topography in the optic nerve

Despite conflicting evidence, the concept of retinotopic arrangement gained increasing support in the early 20th century and was duly extrapolated to the human optic nerve [8]. Two particularly influential articles published in the early 1960s included figures to emphasise this concept [9, 10]. Retinotopic arrangement of the human optic nerve has subsequently been accepted as an established fact and perpetuated in anatomical [49] and medical [50] textbooks, as well as in many expert reviews of the visual pathways published in medical journals [45–47]. The established and entrenched nature of this concept was recently emphasised by an article which derived a map of ‘the retinotopic arrangement of the visual pathways’ from a review of published magnetic resonance (MR) tractographic images in the radiological literature [48], even though tractography cannot provide reliable first-hand anatomical evidence of nerve fibre organisation [66, 75].

The current review of the available literature has confirmed that, on average, there are just over one million fibres in each human optic nerve, though there appears to be very large inter-individual variation which is not explained by the effects of aging. However, this review does not support preservation of a topographic map of the retina, particularly in the posterior part of the human optic nerve. As discussed above, a precise map is, in fact, not possible because of the anatomy of retinal nerve fibres entering the optic disc. Allowing for this, it is entirely possible that some degree of topographical arrangement exists in the anterior portions of the optic nerves. However, any evidence that this arrangement is preserved throughout the entire course of the anterior visual pathways in humans is very weak. What evidence there is suggests that nerve fibres intermingle in the posterior portions of the optic nerves, meaning that they are most likely to be randomly arranged at the point at which they join the chiasm. A revised version of Fig. 1 incorporating this revised concept is shown in Fig. 8, below.

Parenthetically, it should be emphasised that a lack of retinotopic organisation in the anterior visual pathways has no bearing on the well-established retinotopic arrangement of the LGN and visual cortex [44].

### The optic chiasm

#### Introduction

Many excellent reviews have been written about the optic chiasm and its history [76, 77]. In brief, the existence of the chiasm has been known about since at least the time of Hippocrates, but the original concept, as espoused by Galen, da Vinci, Vesalius, Willis, Wren and Descartes, among others, was that the two optic nerves touched each other at the chiasm without interacting before passing backwards to their own sides of the brain. The generation of a single visual percept was considered to occur at a higher level, perhaps in the pineal gland. The possibility that the visual pathways crossed in the chiasm was raised by Albertus Magnus in the 12th century [78] but the first clear description of hemi-decussation, i.e. that nasal retinal fibres crossed while temporal retinal fibres did not, was proposed by Sir Isaac Newton in 1704 [79]. The first diagram depicting hemi-decussation was published a few years later [80], but formal proof had to wait for light microscopy in the 20th century [81].

As discussed above in relation to the optic nerve, conventional wisdom has it that the human optic chiasm is organised topographically such that bundles of nerve fibres from the nasal halves of the two retinae travel backwards to the chiasm in the medial halves of the two optic nerves, cross with their contralateral homologues in the centre of the chiasm, and then leave the chiasm in the medial portions of the two optic tracts as they head for the LGN [Fig. 1]. Meanwhile, the nerve fibres from the temporal halves of the two retinae travel together in the lateral halves of the optic nerves, through the lateral portions of the chiasm and leave the chiasm in the lateral portions of the ipsilateral optic tracts. As discussed above, however, the nerve fibres in the optic nerves are probably not, in fact, arranged in this way when they reach the chiasm. This then begs the question of exactly how the optic nerve fibres are arranged as they pass through the human optic chiasm. Essentially there are four anatomical considerations:

1. are nerve fibres retinotopically organised as they enter the human optic chiasm from the optic nerves?
2. does the centre of the human chiasm contain the nerve fibre crossings, or are the fibres arranged in parallel at that point?
3. which individual nerve fibres cross which other nerve fibres, exactly where in the human chiasm do they cross, and at what angle do they cross?
4. are nerve fibres retinotopically organised as they exit the optic chiasm into the optic tracts in humans?

These issues will be dealt with in turn.

#### Retinotopic organisation at the entrance to the human optic chiasm

The accepted view is that crossed and uncrossed fibres remain distinct from each other in the optic nerve, are retinotopically organised, and diverge only once they have entered the chiasm [44] [Fig. 1]. However, as discussed above, both crossed and uncrossed fibres are probably widely distributed across the entire optic nerves by the time they reach the optic chiasm in humans. The septa in the anterior optic nerves are no longer present at this point, the nerve fibres are no longer arranged in fascicles, and electron microscopy demonstrates clear evidence of lateral movement of nerve fibres in humans [15, 16]. In addition, retrograde studies in monkeys using tracers injected into the eye [66] or optic tract [59] have shown that crossed and uncrossed fibres in the anterior chiasm are scattered quite widely, suggesting that preservation of a retinotopic arrangement at this point is very unlikely.

#### Fibre arrangement in the centre of the human chiasm

It should be emphasised that it is not possible to extrapolate information from most species other than, possibly, non-human primates to humans because of the considerable inter-species difference in anatomy. Apart from anything else, the percentage of fibres which cross depends on how anteriorly or laterally the eyes of a particular species are placed [67], generating a relationship that has been termed the Newton-Müller-Gudden Law [82, 83]. It is generally assumed that about 53% of human optic nerve fibres cross in the human optic chiasm [81].

Beyond this, there is still significant inter-species difference. In the quokka (a marsupial), for example, uncrossed fibres are completely restricted to the temporal parts of the chiasm by a fissure running along its entire length [64, 69]. The chiasm of the tree shrew shows similar segregation [65]. In other animals such as the mouse [84] and ferret [85], there is marked intermingling of crossed and uncrossed fibres within the chiasm. Evidence from old world monkeys suggests that uncrossed fibres remain in the lateral portions of the chiasm [9, 59] but there might still be differences between these and other non-human primates and humans.

The precise arrangement of fibres as they travel through the human chiasm remains uncertain. Over a century ago, Henschen [61], Pick [62] and Wilbrand & Saenger [86] applied the concept of retinotopy to nerve fibres passing through the chiasm even though Dean & Usher [63] had contemporaneously provided clear evidence that crossed and uncrossed pathways overlapped each other to a considerable extent at this point. A subsequent summary by Hoyt suggested that the evidence for a topographical arrangement within the chiasm “contained more speculation than fact” [87]. Nevertheless, as with the optic nerve, conventional wisdom adopted the concept of retinotopy and applied it to the chiasm [Fig. 1]. Additional support for this came from a study involving a series of macroscopic dissections of the chiasm which appeared to show crossing (nasal) fibres travelling in the medial halves of the two optic nerves, fanning out as triangles within the chiasm, and then interdigitating with each other in the centre of chiasm [88]. Overall, however, it is difficult to know how much evidence macroscopic dissections like this can provide about the microscopic anatomy of the nerve fibres.

**Fig. 7:**
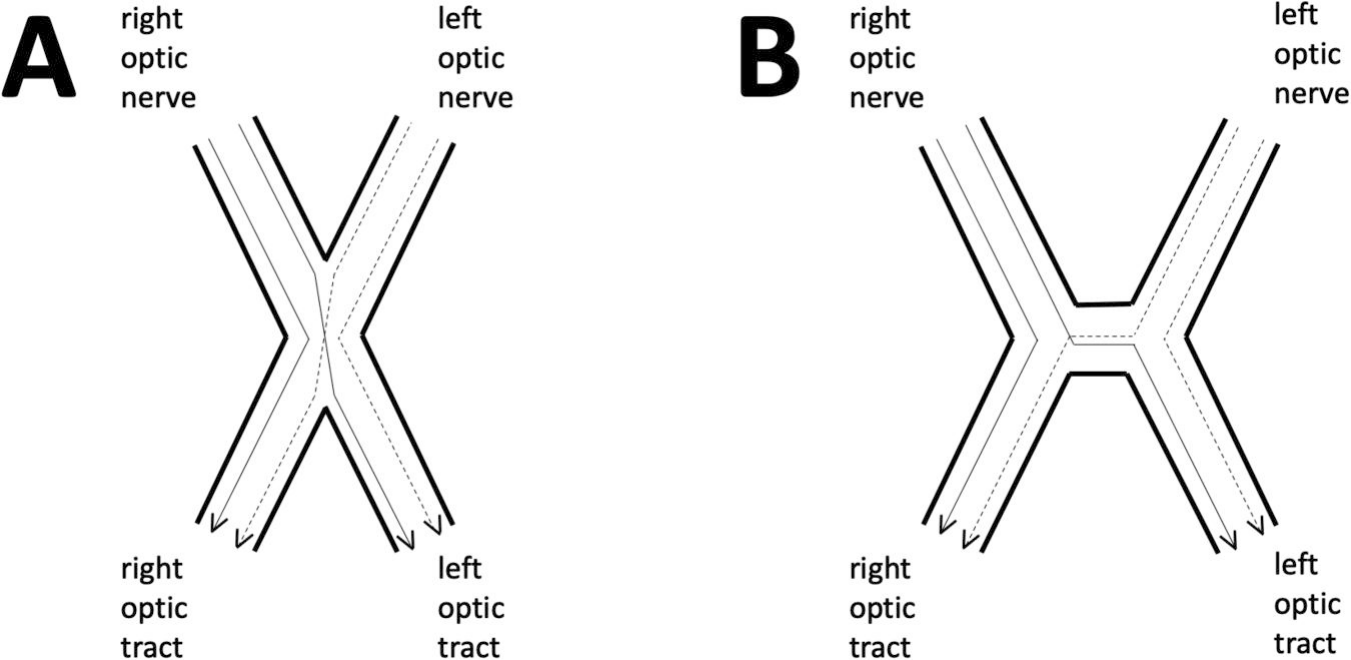
Diagram to clarify the difference between an ‘X-shaped’ and an ‘H-shaped’ chiasm. **A** Nasal fibres cross each other in the centre of an X-shaped chiasm. **B** In contrast, nerve fibres in the crossbar of an H-shaped chiasm are arranged in parallel, and nerve fibre crossings occur paracentrally rather than in the centre.

Fibres destined for the contralateral LGN must pass through the centre of the optic chiasm to get there. However, there are, broadly, two possible arrangements of nerve fibres at the centre of the chiasm, as shown in Fig. 7. The first possibility, and the one that is generally depicted in anatomical textbooks, is that crossing fibres interdigitate and cross each other in the centre of the chiasm with the result that the chiasm can be considered to have the shape of a letter X. The alternative is that individual fibres cross each other paracentrally, i.e. lateral to the centre of the chiasm, before passing in parallel through the geometric centre of the chiasm. These fibres would then cross further fibres on their way to the contralateral optic tract with the overall result that the chiasm would be shaped more like a letter H.

To date, there have been very few published studies looking at the histology of the human optic chiasm. Some studies have reported histological evidence of interdigitation of nerve fibres at the centre of the chiasm in humans [16, 89] but this is not a consistent finding. For example, the histological images of the human chiasms presented in Horton’s review of Wilbrand’s knee (see below) clearly show fibres travelling in parallel along a crossbar at the centre of the chiasm [90]. A recent photomicroscopic study looking at an entire normal human optic chiasm also found that fibres in the centre of the chiasm were arranged in parallel, running in a coronal direction instead of crossing each other at that point [11]. Rather than being located in the centre of the chiasm, nerve fibre crossings occurred paracentrally, i.e. more laterally than was previously realised [11]. This arrangement is, incidentally, consistent with that found in a recent study of the macaque optic chiasm [66].

Evidence from other sources also supports the fact that, in humans, the fibres travelling from one optic nerve to the contralateral optic tract actually travel transversely across the centre of the chiasm (i.e. from left to right and vice versa) rather than diagonally across it. Specifically, multiple MR studies using diffusion tensor imaging (DTI) have suggested a coronal orientation of the nerve fibres in the centre of the chiasm [75, 91–98]. Though compelling, and despite arguments to the contrary [99], it must be remembered that extrapolation from DTI studies to microscopic anatomy of nerve fibres is not possible, bearing in mind the actual size of an individual voxel: within any voxel there are many individual nerve fibres which could be running in multiple different orientations [97]. As things stand, the resolution of MR imaging remains too low to demonstrate decussation of individual nerve fibres reliably and so this technique cannot confidently demonstrate the fine details of intrachiasmal nerve fibre organisation [66, 75].

The idea that fibres cross paracentrally and then travel in parallel through the centre of the chiasm was also supported by a recent MR imaging study looking at the *macroscopic* outline of the optic chiasms of individual people without involvement of their visual pathways. This study found evidence of a crossbar of variable length in all chiasms, consistent with an H-shape [100]. In summary, it is not yet possible to provide an incontrovertible statement, but most evidence suggests that the chiasm is actually H-shaped, not X-shaped, and that any evidence for retinotopic organisation of nerve fibres within the chiasm is, at best, weak.

#### Locations and angles of nerve fibre crossings

As above, recent evidence has suggested that, in humans, most nerve fibre crossings in the optic chiasm are located paracentrally and not at its centre. The majority of nerve fibres appear to cross each other at approximate right angles at this point [11]. This particular study found that inferior fibres crossed more anteriorly in the chiasm while superior fibres crossed more posteriorly: [11] this finding is discussed below in relation to providing an explanation for junctional scotomas. As detailed as this study was, however, an important qualification is that it only looked at microscopic sections in the plane of the chiasm so it could not comment on whether or not there may have been any vertical movement of nerve fibres (and therefore any vertical crossings) as they passed through the chiasm [11].

Again, and contrary to conventional wisdom, Jain et al. [11]. found that, though the nerve fibres travelling in the lateral parts of the chiasm were largely arranged in parallel, there was no clearly demarcated boundary between these fibres and the fibres which were destined to cross to the contralateral optic tract [11]. They also found clear evidence of nerve fibre crossings in the lateral parts of the chiasm, though these fibres crossed each other at lower angles than those in the paracentral regions. Nevertheless, it was clear that some ‘uncrossed’ fibres were crossing ‘crossed’ fibres in the lateral portions of the chiasm.

Precise details are not yet available regarding the angles at which individual nerve fibres cross each other but, overall, the average angle of nerve fibre crossing appears to decrease between paracentral and lateral regions. As above, the extent (both in terms of number and angle) of any vertical crossings between nerve fibres in the human chiasm remains unclear.

#### Retinotopic organisation at the exit from the chiasm to the human optic tract

A study that used polarised light to look at the human optic tract suggested that all fibres were travelling in parallel at this point [101]. Another study of cadaveric human optic tract found that neurons were organised by size, the larger axons being located more ventrally and more superficially in the tract [102]. The latter authors postulated that this meant the fibres were segregated according to whether they originated from X- or Y-ganglion cells in the retina, an arrangement which would obviously not be compatible with a single retinotopic map. These human studies are consistent with animal studies which have also suggested that nerve fibres in the monkey optic tract are organised by fibre size, not retinal location [103, 104].

Other histological studies on non-human primates have similarly concluded that nerve fibres are intermingled rather than being in binocular registration in the optic tracts, meaning that a hemiretinal map (i.e. retinotopic organisation) is unlikely [103, 105, 106]. A more recent study in the macaque found evidence of ‘extensive intermingling’ of crossed and uncrossed fibres in the posterior parts of the chiasm as they exited into the optic tracts [66]. These findings are consistent with the observation of nerve fibre crossings in the lateral parts of the human optic chiasm in the study by Jain et al. [11]. Overall, what evidence there is suggests that it is highly unlikely that retinotopic organisation is preserved by the time optic nerve fibres reach the optic tracts in humans.

### Other considerations

As above, existing evidence does not support the current view that optic nerve fibres are retinotopically organised as they pass through the human optic chiasm. It appears more likely that nerve fibres are more diffusely organised as they enter and exit from it. During the course of this review, information relating to several other topics relevant to the human optic chiasm was uncovered, and these topics will be discussed in this section. Specifically, brief comment will be made on:

1. the clinical finding of binasal visual loss.
2. the existence (or not) of Wilbrand’s knee.
3. the precision of the cut-off in the vertical midline.
4. the existence of retrograde (centrifugal) fibres, i.e. are there any fibres travelling ‘backwards’ from the brain to the retina in humans?
5. what additional nerve fibres (i.e. those not destined for the LGN) travel through the human chiasm?

#### Binasal visual loss

The literature contains small numbers of clinical case reports of binasal visual field loss. This has often been attributed to damage to the laterally placed, uncrossed fibres from, for example, a calcified or dolichoectatic internal carotid artery [107, 108]. However, in several recent cases the patient’s imaging was found to be entirely normal, and the mechanism of the visual loss was thought to be functional, or non-organic [108–110]. These more recent cases cast significant doubt on the earlier reports. Moreover, a recent study looking specifically at visual field loss resulting from eccentric chiasmal compression by pituitary tumours found that disproportionate temporal field loss persisted with eccentric compression and that more lateral compression of the chiasm did not, in fact, generate the corresponding increase in nasal visual field loss that would have been expected with a precise retinotopic arrangement of nerve fibres [111]. Instead, the findings implied that the observed selective vulnerability of crossing fibres was due to factors other than their anatomical arrangement.

#### Wilbrand’s knee

Lesions of the anterior chiasm where it is joined by the optic nerve clinically give rise to a pattern of visual field loss known as a ‘junctional scotoma’ [112]. This consists of central visual loss in the eye whose optic nerve is affected along with visual loss in the upper temporal quadrant of the other eye. An apparent explanation for this phenomenon was provided by Wilbrand in 1904 [90] who described fibres from the inferior nasal retina looping forward into the contralateral optic nerve before they continued their journey posteriorly into the contralateral optic tract [Fig. 1]. Damage to this loop of fibres (subsequently known as ‘Wilbrand’s knee’) neatly explained the visual loss in the eye contralateral to the affected optic nerve. Thereafter, the existence of Wilbrand’s knee was widely accepted [8], becoming established in anatomical textbooks [113] as well as in general medical, neurological, and ophthalmological teaching [114]. Some ‘indirect’ support for its existence was provided by anisotropic light imaging [115], but it is likely that these results were confounded by artefact [116, 117].

Subsequent, careful analysis has not supported the existence of Wilbrand’s knee. Studies of both normal monkeys and humans have clarified that what Wilbrand saw was almost certainly an artefact of enucleation [90]. Similarly, a careful study of three patients whose optic nerves were sectioned precisely at their junction with the optic chiasm demonstrated no evidence of contralateral visual field loss [118]. More recently, a histological study of macaque anterior visual pathway showed no evidence of Wilbrand’s knee in their Fig. 1 [66]. Accordingly, the concept is now considered to have been debunked.

Having said this, an explanation is still required for the clinical observation that compression in the very anterior portion of the chiasm results in a junctional scotoma [112, 119, 120]. An alternative explanation for this phenomenon was recently suggested by Jain et al. [11] who observed that inferior nasal fibres (presumably those subserving the upper temporal visual fields) crossed more anteriorly in the chiasm, unlike the superior nasal fibres which crossed more posteriorly. A similar arrangement was, incidentally, found by Glaser [76] and has also been reported in the macaque [59]. This arrangement could explain the junctional scotoma: a lesion of the anterior chiasm at the point where it is joined by an optic nerve could result in selective visual field loss of the superior temporal field of the contralateral eye (in addition to visual field loss in the ipsilateral eye) because the nerve fibres originating from the contralateral inferior nasal retina of one eye cross just behind the entrance of the other optic nerve.

#### The midline vertical cut-off

The proportion of fibres crossing in the chiasm varies between species as a function of the extent of the binocular visual field and this, in turn, depends on how laterally the eyes are positioned in the head, a relationship which has been termed the Newton-Müller-Gudden Law [82, 83]. It has been pointed out that, in animals with forward-facing eyes such as humans, stereopsis in the vertical midline would be severely compromised if there were an absolute division between left and right hemifields and their respective cortical representations. Were this the case, processing of any binocular disparity of objects whose images straddled the midline would require comparison of information via the corpus callosum, something that would take considerable time and, potentially, present an evolutionary disadvantage [83]. Accordingly, several studies in non-human primates have looked at retrograde labelling in the retina following lesioning [121] or injection of HRP [122] into the optic tract and/or LGN. These studies have consistently found evidence of a small overlap of the retinal ganglion cells projecting ipsilaterally and contralaterally along a narrow vertical band centred on the fovea. This band is about 2°–3° wide at the fovea but narrows to about 1° wide immediately above and below it before progressively widening with increasing eccentricity to about 2°–5° at 5 mm from the fovea and a maximum of 8°–15° peripherally [121–124]. A careful clinical study of hemianopic visual field loss in humans reported evidence of an overlap of similar dimensions [125], as did a study of stereoacuity involving disparity across the vertical midline [83]. Further indirect evidence arguing against a strict midline cut-off comes from an experiment demonstrating that division of the corpus callosum in monkeys did not interfere with midline stereoacuity [126].

In clinical practice, however, this band of overlap is so narrow that it would be difficult to detect on standard perimetry and it certainly cannot explain the clinical phenomenon of macular sparing which requires preservation of at least 2° of central vision [127]. Indeed, a recent detailed review of macular sparing strongly suggests that there are methodological issues which cast doubt on many of the earlier clinical studies looking at macular sparing, concluding that the most likely explanation relates to the anatomy of the blood supply of the occipital lobe [127]. The review provides very strong evidence refuting any explanation involving direct input from ipsilateral retinal ganglion cells in humans [127]. In summary, whether or not any fibres from the nasal retina pass directly through the chiasm to the ipsilateral optic tract in humans remains an unanswered question.

#### Retrograde (centrifugal) fibres

Small numbers of retrograde fibres (also referred to as corticofugal, centrifugal, or ‘efferent’ fibres) have been demonstrated in the optic nerves of most non-human species, potentially arising from eight different locations in the brain [128, 129]. Of course, any fibre which arises in the brain and passes through the optic nerves must also pass through some part of the chiasm. Retrograde fibres appear to be less common in mammals than in lower-order animals [130, 131], though there have been occasional reports in monkeys [132, 133], in whom some fibres destined for the retina appear to arise near the superior colliculus [134]. It has been suggested that, in non-human primates, these retrograde fibres account for about 1% of the nerve fibres in the anterior visual pathways [132].

Though the entire existence of retrograde fibres in the human anterior visual pathway has been challenged [87], some early studies looking at lesions of the human optic nerve suggested that they might account for up to 10% of all optic nerve fibres [135–137]. These studies found that some neurons appeared to survive in the optic nerve after enucleation, the surviving neurons demonstrating stumps whose anatomy and orientation implied attempted centrifugal growth [136, 138–140]. Similarly, a pathological study of a girl with congenital cystic eyeballs showed residual myelinated nerve fibres in the optic nerves which appeared to originate in the hypothalamus; the number of these presumed retrograde nerve fibres was again estimated to be about 10% of a normal optic nerve [137]. A small number of additional studies looking at the human retina have also reported retrograde axons, thought to have originated in the posterior hypothalamus and dorsal raphé nuclei [133, 141–143].

Overall, however, very few studies have reported retrograde fibres in the human optic nerve, and most of those published half a century ago have not been replicated. Bearing in mind the possibility of negative publication bias, it seems sensible to keep an open mind about the presence (or absence) of these fibres in the human optic nerve and chiasm. If they do exist, they could have a role in controlling retinal sensitivity, thereby mediating selective attention [131, 144]. However, their precise number, their route and their role remain undefined.

#### Fibres destined for structures other than the LGN

The vast majority of nerve fibres in the anterior visual pathway are destined for the LGN. However, many other parts of the brain have roles which require direct visual input. It is possible that optic nerve fibres destined for these regions are entirely independent of those destined for the LGN or, alternatively, that some of the other structures are supplied by branching axon collaterals. Nevertheless, in the process of supplying input to both sides of the brain, retinal fibres passing through the chiasm must reach both optic tracts, meaning that individual fibres will either cross to the contralateral optic tract or remain uncrossed (in a similar manner to those fibres destined for the two LGNs). Presumably, any fibres that cross will behave in a similar manner to those destined for the contralateral LGN.

The main additional areas receiving direct visual input are the suprachiasmatic nuclei (SCN, involved in generating and maintaining circadian rhythm), the pretectal nuclei (which mediate the pupillary light reflex), the superior colliculi and accessory optic system (AOS, involved in generating saccades, optokinetic nystagmus, and other eye movements) and the reticular formation (controlling arousal) [47].

Animal studies have shown that the SCN receives direct visual input, including that arising from intrinsically photosensitive retinal ganglion cells [145, 146]. Fibres destined for the SCN travel in the optic nerve and reach it either directly from the optic chiasm or via the optic tract [147]. Input to the SCN has usually been found to be bilateral, meaning that some fibres cross in the chiasm [148]. In rats and hamsters, there is clear evidence of bifurcation of axons destined for the LGN, one branch heading to the SCN [149, 150] but it is not clear whether or not this arrangement applies in humans. Evidence for direct optic nerve fibre input to the SCN in humans comes from an autopsy study of individuals who sustained optic nerve damage during life: degenerating axons were found in the SCN on both sides of the brain, implying that each SCN receives input from both ipsilateral and contralateral eyes [151].

Studies in non-human primates suggest that the projections from the eyes to the pretectal nuclei are largely contralateral [152]. In humans, a careful study of pupillary responses to maximal light stimulation in subjects with lesions of the optic tract demonstrated that between 54%-67% of fibres destined for the pretectal region were crossed [153].

The superior colliculi are involved in the generation of saccadic eye movements while the AOS (the anterior and posterior pretectal nuclei and the nucleus of the optic tract) is concerned with generating optokinetic nystagmus, visual-vestibular interaction and visuomotor coordination. All these centres require, and receive, considerable visual input: it has been estimated that 1%-6% of all visual fibres project to the AOS in non-human primates [154]. The projection to the superior colliculi is bilateral [155]. That to the AOS is predominantly crossed [152, 156, 157] though the proportion of fibres projecting ipsilaterally to the AOS varies between studies, ranging from small numbers to equal numbers of ipsilateral and contralateral projections [154, 158–161]. Information regarding the innervation of the superior colliculi and AOS in humans is lacking.

In summary, an average of about 2 million optic nerve fibres pass through the human optic chiasm. The majority of these are destined for the two LGNs but perhaps 5% are destined for other structures in the brain, including the SCN, the pretectal region, and structures involved in the control of eye movements. While all these pathways might differ somewhat regarding the proportion of crossed and uncrossed fibres, it does appear that a reasonable working assumption is that most fibres not destined for the LGN behave in a similar manner to those destined for the LGN: about half cross to the contralateral side of the brain as they pass through the optic chiasm while the other half do not.

## Discussion

This study has extracted and summarised the information that is currently available regarding the microscopic anatomy and nerve fibre arrangement of the human optic nerve and chiasm. It has confirmed that an average of just over 1 million fibres travel in each optic nerve, though the actual number is highly variable between individuals, the normal range extending between about 500,000 and 1,500,000 fibres/nerve. As expected, most of the nerve fibres in the optic nerves are destined for the LGN but a small proportion, perhaps 5%, are destined for other structures in the brain. While the vast majority of the nerve fibres in the optic nerve are myelinated, a very small percentage are not.

Consistent with accepted teaching, almost all the nerve fibres destined for the ipsilateral and contralateral LGNs are divided by a line running vertically through the retina and centred on the fovea. Existing evidence suggests that there is, in fact, a very narrow vertical band of overlap (i.e. adjacent ganglion cells in this region can project to either LGN) a few degrees wide and centred on the fovea. However, this band of overlap cannot explain the clinical finding of macular sparing. A few other conclusions can be drawn, namely that the existence of Wilbrand’s knee has been firmly debunked and that there is an alternative credible explanation for junctional scotomas [11].

**Fig. 8:**
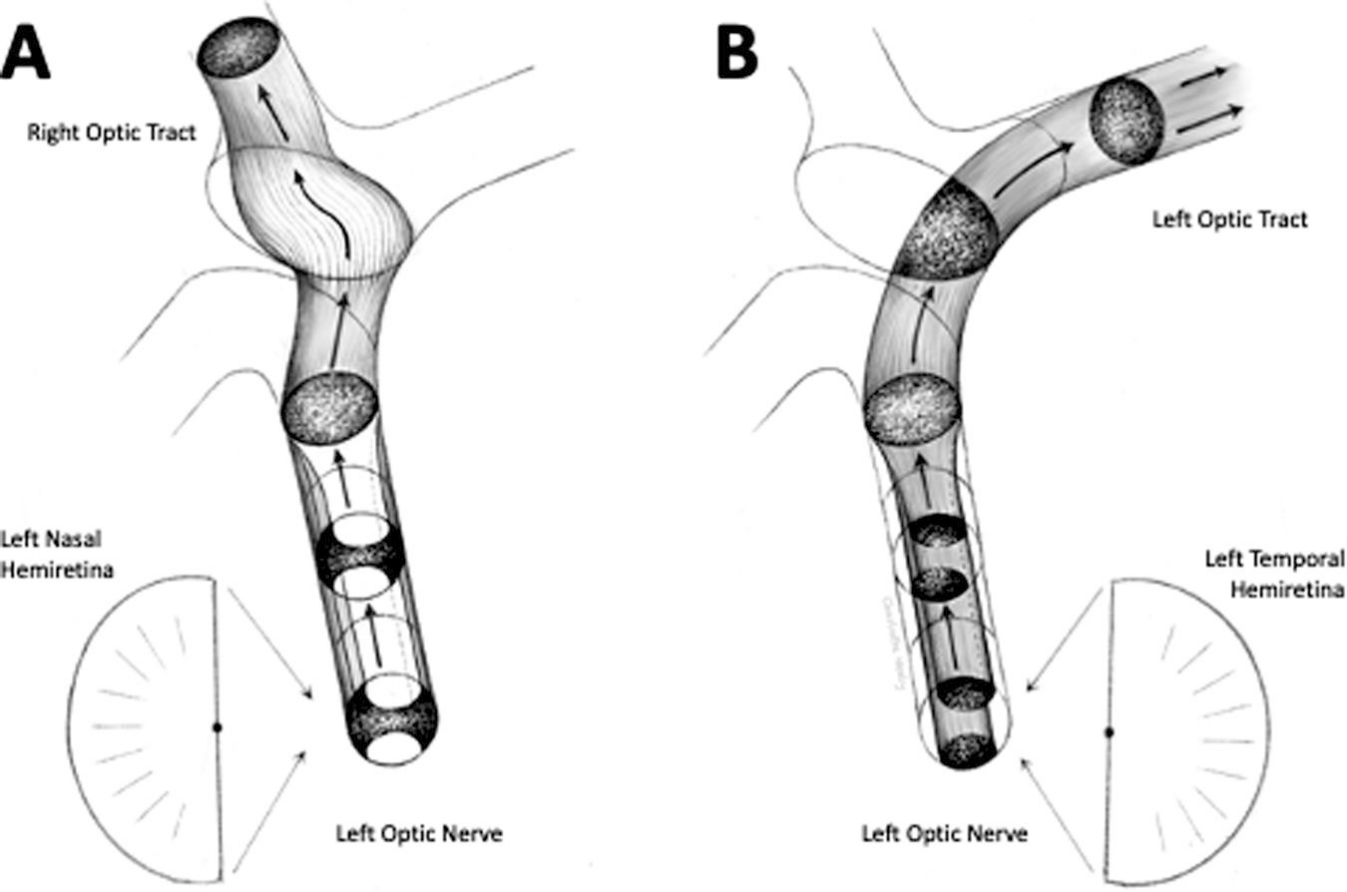
Proposed revision of nerve fibre anatomy in the human optic nerve and chiasm. Fibres arising from the temporal retina are arranged in two bundles separated by fibres arising from the nasal retina (see Fig. 6). As they travel posteriorly in the nerve, all fibres gradually mingle with each other so that they are intermingled by the time they reach the chiasm. **A** Fibres from the nasal retina then travel in parallel along the ‘cross-bar’ of the chiasm to reach the contralateral optic tract. **B** fibres from the temporal retina pass directly backwards to the ipsilateral optic tract.

Information specifically relating to the microscopic anatomy of the optic nerve and chiasm in humans is very limited, but what evidence there is suggests that the precise topographical arrangement of the anterior visual pathway, as currently detailed in many textbooks and review articles, is not preserved. There is reasonably strong evidence that the temporal fibres are arranged in two bundles in the anterior portion of the optic nerve [54]. However, even this arrangement is likely to be lost in the intracranial portions of the optic nerves. Contrary to accepted teaching, optic nerve fibres appear to be diffusely organised and intermingled as they enter and leave the human optic chiasm. This means that the information in many contemporary texts is misleading. Future detailed anatomical studies will undoubtedly provide more clarification but, in the meantime, this review suggests that it would be better to avoid excess detail in the absence of adequate evidence, meaning that existing diagrams of the chiasm should be modified along the lines of Fig. 8.

Available evidence confirms that just over 50% of the nerve fibres cross to the contralateral optic tract in the human chiasm. At a microscopic level, the actual crossings of individual nerve fibres appear to occur paracentrally, not in the geometric centre of the chiasm as has been previously supposed. Several independent sources of information suggest that nerve fibres in the centre of the chiasm are actually arranged in parallel, travelling in a coronal direction. This means that the chiasm is more accurately described as ‘H-shaped’, not ‘X-shaped’. Because of the diffuse distribution of the nerve fibres as they enter and leave the chiasm, it is likely that all nerve fibres cross many other fibres on multiple occasions and that this applies to both ‘uncrossed’ fibres destined for the ipsilateral LGN as well as to ‘crossed’ fibres heading contralaterally. Having said this, it is likely that the ‘crossed’ fibres will cross larger numbers of other individual nerves than the ‘uncrossed’ fibres before reaching their target optic tracts because their journey is longer.

Returning to the finite element model of chiasmal compression referred to in the introduction, the outcome of this review means that the original study [7] was, unfortunately, based on incorrect assumptions about the microscopic arrangement of nerve fibres in the chiasm. Having said this, nerve fibres which eventually reach the contralateral LGN cross more nerves than those heading ipsilaterally, and their crossings probably occur at higher angles, at least on average. This means that the ‘crossing hypothesis’ may still help explain the increased vulnerability of crossing fibres but the supporting evidence from finite element modelling needs re-evaluation. It is the authors’ opinion that, if the crossing hypothesis can be shown to be relevant in relation to the optic chiasm, the concept may have more wide-reaching implications for pathological compression of nerve fibres at other sites, for example, in peripheral nerves, the brain and the spinal cord. Accordingly, further work is currently underway to revise the finite element model by incorporating the updated anatomical information derived from this review.
