# Correction: Nerve fibre organisation in the human optic nerve and chiasm: what do we really know?

**DOI:** 10.1038/s41433-025-03800-7

**Published:** 2025-04-07

**Authors:** Pratap R. Pawar, Joshua Booth, Andrew Neely, Gawn McIlwaine, Christian J. Lueck

**Affiliations:** 1 School of Engineering and Technology, University of New South Wales, Canberra, NSW Australia; 2 School of Medicine and Psychology, Australian National University, Canberra, NSW Australia; 3 Department of Ophthalmology, Mater Hospital, Belfast, Northern Ireland

**Keywords:** Retina, Object vision

Correction to: *Eye* 10.1038/s41433-024-03137-7, published online 07 June 2024

Several of the background arrows in figure 6 were incorrectly positioned. These must have moved during the preparation of the figure for submission. Unfortunately, this was not picked up during the correction process.

The original article has been corrected.

